# Unusual Ether Lipids and Branched Chain Fatty Acids in Sea Cucumber (*Cucumaria frondosa*) Viscera and Their Seasonal Variation

**DOI:** 10.3390/md20070435

**Published:** 2022-06-29

**Authors:** Reem Abuzaytoun, Suzanne M. Budge, Wei Xia, Shawna MacKinnon

**Affiliations:** 1 Department of Chemistry and Physics, Mount Saint Vincent University, Halifax, NS B3M 2J6, Canada; 2 Department of Process Engineering and Applied Science, Dalhousie University, Halifax, NS B3H 4R2, Canada; suzanne.budge@dal.ca; 3 Mara Renewables Corporation, Dartmouth, NS B2Y 4T6, Canada; wxia@maracorp.ca; 4 Agriculture and Agri-Food Canada, Kentville, NS B4N 1J5, Canada; shawna.mackinnon2@agr.gc.ca

**Keywords:** diacylglycerol ether, 1-*O*-alkylglyceryl ether, nutraceutical oils, bêche-de-mer

## Abstract

The sea cucumber, *Cucumaria frondosa*, is harvested primarily for its muscular bands and body wall. Development of a nutraceutical product based on lipid recovered from its viscera would give commercial value to the entire organism; however, such development requires knowledge of the lipid and fatty acid (FA) profiles of the viscera. Here, we describe the lipid and FA composition of viscera recovered from *C. frondosa* harvested in coastal waters in the northwest Atlantic, taking into account variation due to harvest season. We found highest lipid content at ~29% in winter, with diacylglyceryl ethers (DAGE) comprising ~55% of the total lipid mass and triacylglycerols (TAG), phospholipids (PL) and monoacylglycerol ethers (MAGE) at 5–25% each. The branched chain FA, 12-methyltetradecanoic acid (12-MTA), represented 42% of total FA mass in DAGE. In summer, lipid content was lower at 24% and TAG was the dominate lipid, with proportions more than double that found in winter (45% vs. 20%); DAGE in summer dropped to ~30% of total lipids. In TAG, 12-MTA was much lower than found in DAGE in winter, at only 10% but eicosapentaenoic acid (EPA) content was ~20%, which brought the total EPA% to 28% of total FA—the highest among all three seasons. There was little effect of season on MAGE or PL proportions. These data can help harvesters maximize catch efforts in terms of lipid yield and profile.

## 1. Introduction

The sea cucumber, *Cucumaria frondosa*, is widely distributed in the cold waters off the coast of the United States and Canada. Along Nova Scotia and New Brunswick, Canada, *C. frondosa* is harvested as a by-catch [1] and, presently, only the muscular bands and body wall have commercial value; the viscera, representing 50% of the sea cucumber biomass, is discarded [2]. In 2018 the sea cucumber fisheries in Newfoundland reported landings of 5500 tons that were estimated to have a $6,000,000 value to the fishery [3]. The lipid extracted from the viscera has recently been identified as a potential source of marine lipids for nutraceutical applications [4]. However, little is known about the lipid and fatty acid (FA) composition of sea cucumber viscera.

Freeze-dried powders of *C. frondosa* harvested off the coast of Newfoundland [4] were reported to contain up to nine lipid classes including hydrocarbons, steryl esters, ethyl ketones, triacylglycerols (TAG), free fatty acids (FFA), alcohols, sterols, and phospholipids (PL). However, other studies of related species in the Holothurian class of sea cucumber, including *C. japonica*, *C. okhotensis*, *C. fraudatrix* and *Stichopus japonicas*, have identified ether lipids, specifically 1-*O*-alkylglyceryl ethers [5,6]. Diacylglyceryl ethers (DAGE) and monoacylglyceryl ethers (MAGE) have been reported in several marine animals, such as in the livers of dogfish and shark [7] and deep-sea squids (*Berryteuthis magister*) [8]. In the last two decades, ether lipids have attracted the interest of researchers due to their health promoting effects in humans, specifically for their potential in cancer therapy but also because they have been used to improve the bioavailability of other lipid molecules such as butyric acid or omega-3 FA [9,10,11,12].

A branched chain FA, 12-methyltetradecanoic acid (12-MTA), has also been reported in *C. frondosa*. For instance, in fresh and rehydrated powders of *C. frondosa* consisting of body walls and internal organs, 12-MTA was present at 4–9% of total FA mass [13]. However, neither Vaidya and Cheema [2] nor Mamelona et al. [4] identified this FA in their evaluation of *C. frondosa*. All three studies did quantify eicosapentaenoic acid (EPA) at proportions ranging from 17 to 52%, with the wide variation due to the type of sample evaluated; the lowest EPA proportion was found in the sample consisting of only viscera. As an omega-3 FA, EPA is well-known for its role in prevention of cardiovascular disease [14], while 12-MTA has been shown to have anti-inflammatory, anti-cancer and wound healing activity [15]. Thus, *C. frondosa* viscera lipids may contain a number of important bioactive components.

Our main objective was to characterize the lipid content and composition of *C. frondosa*. Since most marine animals show seasonal variation in those parameters due to both environmental factors and reproduction cycles [16], we also aimed to determine the differences in major lipid classes and FA profiles. Specifically, we describe here the variation in lipid classes and their lipid-specific FA proportions across three harvesting seasons in a single year with a goal of providing critical information in the development and marketing of sea cucumber viscera and lipids derived from it.

## 2. Results

The total lipid content of the viscera ranged from ~21–29% dwb (dry weight basis) with the winter harvest showing significantly higher levels (ANOVA; *p* < 0.05) than that observed for spring and summer (Table 1). Moisture content was lowest in winter at ~75%. Spring and summer samples were equivalent in terms of lipid and moisture content. EPA was the major PUFA and ranged from ~25–28%, with highest proportions in summer. In total lipids, PUFA varied between 27–30% of total FA mass with highest levels observed in the summer harvest (Table 1). The FA 12-MTA varied in a similar fashion with almost twice the proportion in winter. Total branched chain FA showed greater variation, with levels from 18–31% and largest amounts in winter.

HPLC analysis indicated that viscera lipids consisted predominantly of TAG and DAGE, with their identities confirmed through elution of authentic standards (Supplementary Figure S1). Undifferentiated PL were prominent peaks and eluted with the most polar mobile phase. Minor peaks coincided with retention times of FFA, DAG and MAG standards. TLC analysis largely confirmed the identities of peaks a, b, c and f in the HPLC chromatogram (Supplementary Figure S1), with the presence of four TLC bands corresponding to TAG, DAGE, FFA, and PL. MAG eluted with PL in the TLC analysis. A fifth band, eluting between FFA and PL, could not be conclusively identified as its R_f_ did not match that of any available lipid standards. Based on the presence of DAGE, it was assumed to be MAGE. This also suggested that the ‘DAG peak’ (peak d in Supplementary Figure S1) identified by HPLC was in fact MAGE; however, without a MAGE standard, the identity could not be confirmed, and, thus, NMR and GC-MS analysis were pursued.

**Figure 1:**
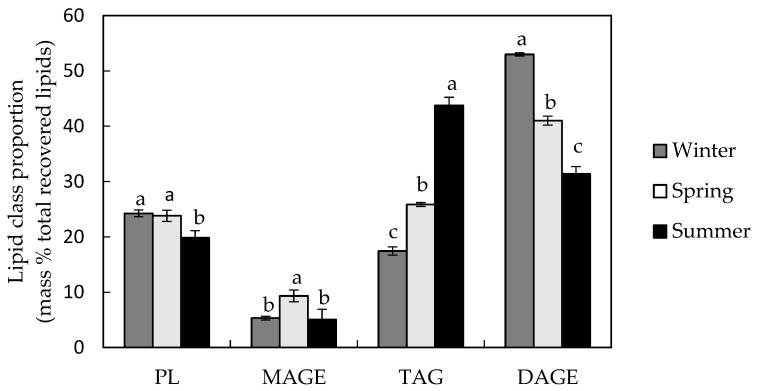
The approximate mass percentage of each lipid class in *C. frondosa* viscera extract relative to total lipids recovered from TLC plates (mean ± SD, *n* = 3). Values with different letters within a lipid class are significantly different (ANOVA; *p* < 0.05). The percentage of FFA was below detection limits. The PL proportion included a small contribution from MAG.

The main lipid class in viscera of sea cucumber harvested in the winter and spring was DAGE which represented ~55% and ~40% of total recovered lipids, respectively (Figure 1), while TAG was the main lipid class found in the summer and represented ~45% of total recovered lipids. For both DAGE and TAG, lipid contents varied by season (ANOVA; Tukey’s test; *p* < 0.05). There was significantly more MAGE in the spring season than the other two (ANOVA; Tukey’s test; *p* < 0.05); PL concentrations were lowest in summer.

**Figure 2:**
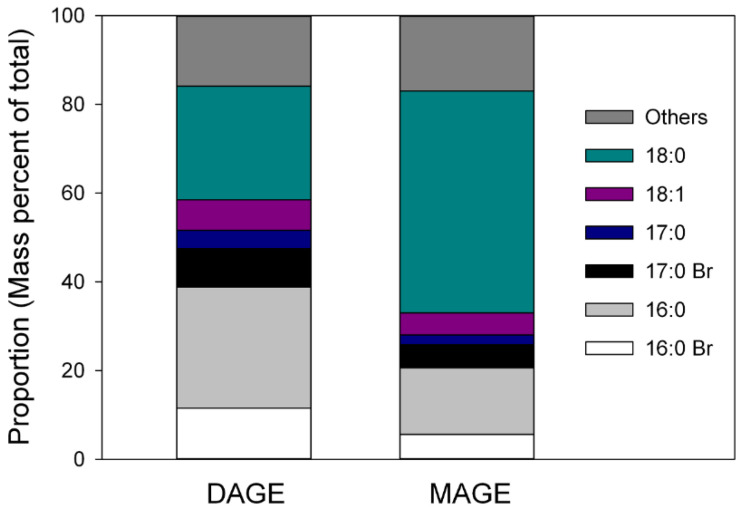
Approximate proportions of major alkyl structures in DAGE and MAGE in viscera lipids of *C. frondosa*.

NMR analysis of the tentatively identified MAGE band contained peaks between 70–75 ppm in ^13^C NMR spectra (Supplementary Figure S2a) and at ~3.5 ppm in the ^1^H NMR (Supplementary Figure S2b) spectra which supported the presence of compounds containing ether bonds [17]. The identity of the MAGE bands was further confirmed using GC-MS analysis of the saponified and acetylated MAGE and DAGE bands and the DAGE standard. Analysis by EI of both of the saponified, acetylated bands yielded TIC with peaks that had similar spectra to the saponified and acetylated 1-*O*-hexadecyl-2,3-hexadecanoyl glycerol standard, indicating that ether structures were likely present. The saponified, acetylated DAGE standard analyzed by CI contained a single component with base peak at *m/z* 341, representing a [M − 59] ^+^ fragment. Based on this fragment, masses and tentative identities were assigned to the other peaks in the two bands. The major alkyl structures were 16:0 and 18:0, with 18:0 dominating in MAGE and roughly equivalent amounts of both in DAGE (Figure 2). Proportions of all other alkyl groups were similar in the two lipids. Seasonality was not evaluated in DAGE and MAGE.

FA composition varied by lipid class and season. The major FA identified in DAGE was 12-MTA, with highest proportions in winter/spring at 41–42% (Table 2). Other prominent branched chain FA included 4,8,12-Me-13:0, 8,12-Me-14:0, and 12-Me-15:0, with levels varying from 2–8% and generally following the same pattern as 12-MTA with lowest amounts in summer. Monounsaturated 16:1n-7 was present in second greatest amounts at ~20% with slightly lower levels in summer. The only prominent polyunsaturated FA was EPA with highest levels of ~9% in summer. In contrast, EPA was the major FA in MAGE in winter/spring at ~62% while 12-MTA was present at only ~5% in all seasons. In total, branched chain FA were present at 8–12% of total FA. In both lipid classes, EPA, branched chain FA, and 16:1n-7 accounted for ~78–85% of the total FA identified, showing very little diversity in structure in the ether lipids.

Prominent FA in TAG were similar to those of DAGE with 12-MTA present at 10–20% with highest levels in winter/spring and total branched chain FA at 17–31%, also with the highest amount in winter/spring (Table 3). Both 16:1n-7 (24–28%) and EPA (14–20%) had greatest levels in summer. Proportions of FA in PL were different than the other three lipid classes. Branched chains FA were only present at 1–5% with 12-MTA < 1%. Instead, straight chain saturates, 16:0, 18:0 and 20:0, became much more important with total saturated FA at 11–19% with highest levels in winter. For the monounsaturates, 18, 20, 22 and 24 carbon FA were present at 1–4% each so that the total for the group ranged from 16–20% (highest in summer); 16:1n-7 only represented ≤ 3% total FA. EPA was the dominant FA at 43–55% with a maximum in winter/spring and DHA was present at highest proportions in all lipid classes at ~1.5%, also with a high in winter/spring.

## 3. Discussion

The presence of ether lipids in *C. frondosa* viscera was expected as alkyl structures including saturated (branched and unbranched) and monounsaturated alkyl chains associated with an ether bond in glycerol ethers have been previously identified in Holothurians [5,6,18]. High levels of α-glyceryl ethers have been reported in lipid extracts of *Stichopus japonicas* (18%), *C. fraudatrix* (9.3%), and *C. japonica* and *C. okhotensis* (25–27%) [5,6]. The presence of DAGE ether lipids in other marine species including, for example, dogfish, shark, deep-sea squids, elasmobranch fish, oysters, sponges, snails and corals [7,8,19,20,21,22,23,24] suggests that they are ubiquitous in marine animals and therefore their presence in sea cucumber species such as *C. frondosa* is not surprising; however, total DAGE and MAGE detected here in winter was >55%, exceeding even that reported in some species of shark liver oil [25] and representing a valuable source of non-polar ether lipids. Notably, the total ether lipid level is likely even higher than we have reported since we did not attempt to quantify polar ether lipids.

Analysis of the components of glyceryl ethers derived from both DAGE and MAGE revealed unique alkyl chain structures that were characteristically rich in 16:0 and 18:0 (Figure 2). Other studies have found a similar dominance of 16 and 18 carbon *O*-alkyl chains in DAGE and MAGE of *C. japonica, C. okhotensis* and *Oneirophanta mutabilis* [5,18]. This narrow range of alkyl structures indicates a high specificity for particular substrates in the pathways responsible for the biosynthesis of alkylglycerols [26]. Rybin et al. [5] and Santos et al. [18] had suggested that the specificity might be due to two acyl-CoA reductase isoenzymes (FAR1 and FAR2) [27,28]. Both reductase isoenzymes select fatty acyl-CoA of 16 and 18 carbon chains; FAR2 is specific for palmityl-and stearyl-CoA substrates, while FAR1 targets monounsaturated palmitoleyl- and oleyl-CoA and polyunsaturated linoleyl-CoA [29]. The specificity of these enzymes was first identified in mammalian cells, but our data suggest that they likely exist in sea cucumbers as well.

The FA profile for DAGE and MAGE were characterized by the presence of high concentrations of 12-MTA and EPA, respectively (Table 2). The patterns between the alkyl groups and FA associated with DAGE and MAGE were therefore quite different. In the biosynthesis of ether lipids, 1-*O*-alkyl-*sn*-glycero-3-phosphate has been reported to be a glycerol-based intermediate found mostly in mammalian cells [30]. Esterification of this intermediate with acyl-CoA at the *sn*-2 position in the presence of alkyl-acyl-glycero-3-phosphate acyltransferase results in the formation of 1-*O*-alkyl, 2-acyl-*sn*-glycero-3-phosphate. Removal of the phosphate group from the sn-3 position of that structure by phosphohydrolase results in the formation of 1-*O*-alkyl-2-acyl-*sn*-glycerol (MAGE). DAGE is then synthesized when 1-*O*-alkyl-2-acyl-*sn*-glycerol is esterified with a long-chain acyl-CoA ester by an acyltransferase. Thus, the high concentration of EPA in MAGE is likely due to a high specificity of alkyl-acyl-glycero-3-phosphate acyltransferase in positioning long chain FA (EPA) at *sn*-2 position. Since an elevated level of EPA is not observed in DAGE, that lipid cannot be derived from simple acylation of MAGE; another mechanism must be involved and responsible for the hydrolysis of EPA in MAGE and replacement with 12-MTA in DAGE. It may be that the MAGE we found represent a deacylation product of DAGE, rather than an intermediate in DAGE synthesis. In that scenario, the prominence of EPA would suggest that DAGE containing EPA in the *sn*-2 position are the primary substrates for diacylation at the sn-3 position. Given the similarly high levels of EPA in the PL, it could also be that MAGE containing EPA in the *sn*-2 position are destined for PL synthesis while those species without are preferentially converted to DAGE. Regrettably, we did not carry out a detailed analysis of the PL composition to evaluate its ether lipid structures.

The high EPA levels we found in MAGE could also be due to contamination of the MAGE bands with another lipid class, such as DAG, since a peak with the same retention time as DAG was found in HPLC analysis; however, given the similarity in retention time of DAGE and TAG, it is possible that the peak with the same retention time as DAG was MAGE. The TLC analysis supported this, indicating that DAG was not present in our sample, since its R_f_ did not coincide with any bands, including MAGE. We are also confident that the MAGE band recovered from the TLC plate did contain glyceryl ethers since we identified alkyl structures within it. Thus, we believe that we have isolated a relatively pure MAGE band that did not contain contribution from DAG.

Different species of branched chain FA were identified in both DAGE and MAGE lipid classes. *Iso*- and *anteiso* branched chain FA were the most abundant monomethyl branched chain FA. Zhong et al. [13] quantified *ai*-15:0 in fresh and dried *C. frondosa* harvested near Newfoundland but no other branched chain FA were identified. *Iso*- and *anteiso*- branched FA with carbon numbers from 14–18 have been reported in small amounts in lipids extracted from many types of marine fish [31,32]; 4,8,12-Me-13:0, generally assumed to be derived from phytanic acid sourced from phytoplankton, has also been reported in many marine organisms [33]. However, a number of other single- and multi-methylated branched FA, such as 12-Me-15:0, 8,12-Me-14:0, and 8,12-Me-15:0, have not been commonly reported in lipid extracts of marine species in general, and have never been identified in sea cucumber. While 4,8,12-Me-13:0 can be derived from phytol, a product of photosynthesis, the presence of the other FA with methyl branches in the same position on different carbon numbered backbones suggests that it and the other branched chain FA are products of de novo synthesis.

The synthesis of branched chain FA in mammals has been recently described [34] and it was found to follow similar pathways as used by bacteria [35]. For instance, *iso*- and *anteiso*-branched FA can be produced biosynthetically through regular mechanisms for the synthesis of saturated FA (including involvement of acyl carrier protein) with the substitution of different primer molecules (2-methylpropanyl-CoA, 3-methylbutyryl-CoA, and 2-methylbutyryl-CoA) for acetyl-CoA with activation by the same enzyme (FA synthase) [35]. For example, the initial step for the syntheses of 12-MTA (*anteiso*-branched FA) would involve the use of one 2-methylbutyryl-CoA and 5 acetyl-CoA additions. Similarly, 4,8,12-Me-13:0 branched chain FA (an *iso*-branched FA) would involve the use of 2-methylpropanyl-CoA as a primer molecule followed by a combination of two successive 2-methylbutyryl-CoA and one acetyl-CoA additions. Branched chain FA and specifically 12-MTA have been shown to exhibit promising health benefits including anti-inflammatory, anti-cancer activity and wound healing activity [15]. Thus, their de novo synthesis in *C. frondosa* could represent an underutilized source of 12-MTA for applications in medical treatments.

Viscera lipid and FA composition varied with collection season, as commonly found with many other fish and invertebrate species [36,37], likely due to variation in diet. With the seasonal data presented here, it becomes possible to target specific harvests to maximize the yield of particular lipids or FA. For instance, the overall yield of EPA was higher in viscera in sea cucumbers harvested in summer, likely because TAG was the dominate lipid class in that season and contained ~20% EPA, so that if an omega-3 product was desired, viscera from summer harvest would be a better source. However, the more novel characteristics of the viscera are the ether lipids and 12-MTA. Those would be better targeted with a winter harvest when overall lipid yields are highest and DAGE comprising some 55% of total lipids. It is also the viscera from winter harvest that has highest 12-MTA, so its recovery would also be maximized at that time. In the production of nutraceutical oils, PL are often removed through degumming as part of the refining process [38]. By considering the FA composition within lipid classes, it is also possible to predict the effect of degumming on final lipid composition. One would anticipate, for example, that PL removal would result in an oil that would be lower overall in EPA content, but 12-MTA and ether lipids would comprise a greater proportion of the final product, with this effect being greatest in winter harvested animals. Thus, the data presented here can aid in production of both crude lipid from sea cucumber viscera with preferred lipid characteristics and the final purified product.

## 4. Materials and Methods

Sea cucumber viscera were donated by Ocean Pride Fisheries Limited (Lower Wedgeport, NS, Canada) from harvests conducted in January 2015 (winter; latitude 4439/longitude 6041), March 2015 (spring; latitude 4439 longitude 6040) and July 2015 (summer; latitude 4439/longitude 6041) from the Sable Island Banks off Nova Scotia, Canada. Fall harvests were not carried out. Viscera was stored frozen at −30 °C prior to evaluation.

### 4.1. Moisture and Lipid Content

Portions of frozen *C. frondosa* viscera were homogenized using a food processor. The moisture content was determined by drying ~25 g of homogenized viscera at 100 °C [39] to a constant mass. Lipids were determined gravimetrically after extraction following the Bligh and Dyer method [40] with slight modification. Briefly, 200 g of ground viscera were blended with 200 mL of chloroform and 400 mL of methanol. The mixture was filtered and the residual material was placed in a blender, re-extracted with 200 mL chloroform and filtered again. The combined chloroform/methanol extracts were then mixed with 200 mL of aqueous 0.88% potassium chloride and allowed to separate in a separatory funnel. The lower organic layer was filtered through a bed of anhydrous sodium sulfate to remove the residual water in the lipid. The solvent was removed using a rotator evaporator at 40 °C.

### 4.2. Lipid Class Analysis

Samples for lipid class profiling using HPLC were prepared by dissolving 30–35 mg of lipids in 1.0 mL of dichloromethane. A 1.0 μL aliquot of the lipid (30 mg mL^−1^ dichlormethane) was injected into an Agilent 1100 HPLC equipped with an YMC PAK-PVA-SIL-NP column (250 × 4.6 mm I.D.; 5 µm) and an ESA Corona Charged Aerosol Detector (CAD). The column was eluted with a gradient containing 0.2% *v*/*v* ethyl acetate in isooctane (solvent A), 0.02% *v*/*v* acetic acid in 2:1 acetone: ethyl acetate (solvent B) and 0.1% acetic acid in 3:3:1 *v*/*v*/*v* isopropyl alcohol: methanol: water (solvent C), at a flow rate of 1.5 mL min^−1^ over a run time of 77 min with a post-run time of 12 min (see Supplementary Table S1 for full details). Lipid standards (free fatty acid (FFA), monoacylglycerol (MAG), diacylglycerol (DAG), triacylglycerol (TAG), diacylglyceryl ether (DAGE), phospholipids (PL); see Supplementary Materials for identities) prepared at a concentration 1 mg mL^−1^ in dichloromethane were injected to determine retention times of each lipid component.

### 4.3. Isolation of Lipid Classes by TLC

Lipids (100 µL at 250 mg mL^−1^) were streaked using capillary tubes onto a pre-coated silica gel TLC plate (20 × 20 cm, layer thickness 0.25 mm) which had been previously developed in ethyl acetate and activated in an oven for one hour at 100 °C. The streaked plate was developed in hexane: diethyl ether: acetic acid (80:20:1 by volume) and sprayed with 0.2% methanolic 2,7-dichloroflourescein for visualization of the bands upon exposure to ultraviolet light (360 nm). Lipid standards (wax ester (WE), DAGE, TAG, DAG, MAG, FFA, Phosphatidyl choline and cholesterol) were used to tentatively identify the lipid present by comparison of R_f_ values. Non-polar lipid (DAGE, TAG, FFA) bands were scraped off the plate and extracted three times with 3 mL of 1:1 (*v*/*v*) hexane: chloroform. Polar lipids (MAGE, PL) were recovered similarly using 2:1 methanol: chloroform (*v*/*v*). The solvent was evaporated using a stream of nitrogen and the weight of lipid in each band was determined. Recovered simple lipids (MAGE, DAGE, TAG and FFA) were dissolved in hexane, while PL was dissolved in methanol.

### 4.4. ^1^H NMR and ^13^C NMR Analysis

The NMR spectra of lipid classes identified as DAGE and MAGE using TLC were recorded on a Bruker Avance 500 MHz spectrometer. The recovered bands were dissolved in deuterated chloroform (CDCl_3_) at 16 mg mL^−1^ for ^1^H NMR, and 73 mg mL^−1^ for ^13^C NMR analysis before being transferred to NMR tubes (5 mm diameter, 8 in. in length, Wilmad-LabGlass, Vineland, NJ, USA). The solvent residual signal in the CDCl_3_ was used as a reference for chemical shift assignments. Its ^1^H NMR shift was 7.2–7.3 ppm and ^13^C NMR shift is ~77 ppm. The ^1^H NMR acquisition parameters were modified from a previous study [41]: spectral width, 10,080 Hz; relaxation delay, 3 s; number of scans, 32; acquisition time, 3.25 s; total acquisition time of 6.9 min. The ^13^C NMR acquisition parameters were as follows: spectral width, 33,333 Hz; number of scans, 512; acquisition time, 0.81 s; with a total acquisition time of 16.07 min.

### 4.5. Ether Lipid Identification Using GC-MS

DAGE and MAGE bands were saponified to yield glyceryl ether diols according to Christie [42] with slight modifications. The lipid was suspended in 5 mL of 2 M ethanolic potassium hydroxide and the mixture was flushed with nitrogen, sealed and heated at 100 °C for one hour. It was then cooled to room temperature, and 16 mL water and 8 mL of 1:1 hexane: diethyl ether were added to the test tube. The mixture was vortexed and, after separation of layers, the top organic layer containing unsaponifiable material was recovered. The isolated organic layer was washed with 8 mL RO water and evaporated under a stream of nitrogen. The resulting diols were then acetylated by adding 0.5 mL of acetic anhydride and pyridine (5:1) and allowing the mixture to sit overnight at room temperature [43]. The solvent was then evaporated, the remaining acetylated material was dissolved in hexane. The DAGE standard was similarly saponified and acetylated.

Saponified and acetylated recovered MAGE and DAGE were analyzed using a GC Ultra gas chromatograph coupled with a PolarisQ mass spectrometer (Thermo Fisher Scientific Inc., Waltham, MA, USA). The analysis was performed using electron ionization (EI) and chemical ionization (CI) modes with split-injection (1/100) at 250 °C. The separation was performed on a ZB-35 capillary column (35%-phenyl)-methyl polysiloxane; 30 m × 0.25 mm i.d × 0.25 µm film thickness) with helium as the carrier gas at 1.2 mL min^−1^. The initial temperature for the program was held at 180 °C for 1 min, then increased to 350 °C at 5 °C min^−1^ and held for 3 min. The ionization energy used was 70 eV, with a multiplier voltage of 1643 V, source temperature at 200 °C, and transfer line temperature of 350 °C. Spectral data were acquired over a mass range of *m*/*z* 60–600 in both modes and the emission current was 250 µA. In CI mode, methane was used as the reagent gas at a flow rate of 1.5 mL min^−1^. Alkyl groups associated with an ether bond in both DAGE and MAGE lipid classes were identified by ion spectra obtained for peaks occurring at unique retention times and after subtracting 59 (acetoxy group) from the total molecular mass of DAGE and MAGE.

### 4.6. GC-FID Analysis of FAME

Acid catalyzed transesterification with 0.5 N H_2_SO_4_ in methanol was used to produce FAME of recovered lipid classes [44]. FAME were determined using a GC (Bruker, SCION 436-GC) fitted with a DB-23 column ((50%-cyanopropyl)-methylpolysiloxane) (30 m × 0.25 m × 0.25 µm film thickness, Agilent Technologies, Santa Clara, CA, USA) and a flame ionization detector (FID). Splitless injection was used with an injector temperature of 250 °C. FAME samples were separated using the following temperature program: the initial temperature was held at 60 °C for 1 min, then increased to 153 °C at 45 °C min^−1^, held for 2 min, then increased to 174 °C at 2.3 °C min^−1^ and held for 0.2 min, increased to 205 °C at 2.5 °C min^−1^ and held for 2.5 min for a total run time of 41 min.

### 4.7. Analysis of 3-Ppyridylcarbinol Ester Derivatives

Direct transesterification can be used to prepare 3-pyridylcarbinol ester derivatives from lipid samples [45,46]. Potassium *tert*-butoxide in tetrahydrofuran (0.1 mL, 1.0 M) was first mixed with 3-pyridylcarbinol (0.2 mL), and the mixture was added to 10 mg of lipid in 1 mL dry dichloromethane. After mixing, the sample was incubated at 40 °C for 30 min and then allowed to cool. Water (2 mL) and hexane (4 mL) were added, and the contents of the tube were mixed. The upper organic layer was collected, dried over anhydrous sodium sulfate, and evaporated to dryness using a flow of nitrogen. The resulting 3-pyridylcarbinol esters were dissolved in hexane (0.4 mg mL^−1^) and analyzed by GCMS on a DB-1 DB-1ms capillary column (100% dimethylpolysiloxane, 30 m × 0.25 mm i.d. × 0.25 um film thickness) using the same system as described above. Helium was the carrier gas at a flow rate of 1 mL/min with splitless injection (280 °C). The initial oven temperature was held at 60 °C for 2 min, then increased to 235 °C at 20 °C min^−1^, followed by increasing to 280 °C at 2 °C min^−1^ and holding for 10 min. The ionization energy was 70 eV, with multiplier voltage of 1643 V, source temperature at 200 °C, and transfer line at 280 °C. Spectral data were acquired over a mass range of *m*/*z* 60–500. The molecular weight of a 3-pyridylcarbinol ester derivative was determined by the molecular ion, while a 28 amu gap in the EI spectra indicated the location of the methyl branch.

### 4.8. Statistical Analysis

All samples were run in triplicate and all data were analyzed using one-way analysis of variance (ANOVA) with an α level of 0.05 (Minitab 17). Tukey’s multiple comparison test was used when ANOVA indicated there were significant differences in the means of the three harvests (α level of 0.05).

## 5. Conclusions

Five lipid species were identified in *C. frondosa* using HPLC and TLC with structural identification by GCMS when necessary, and included DAGE, TAG, FFA, MAGE and PL. All except FFA were present in sufficient quantities to characterize their acyl and alkyl structures. In winter, DAGE dominated, contributing ~55% of total mass of the lipid extract; 12-MTA comprised 42% of total FA within DAGE. In summer, TAG was present in the greatest amounts at 44% with lower 12-MTA amounts but EPA was more abundant (20% of total FA). Proportions of PL and MAGE showed little variation by season, in comparison to DAGE and TAG. These results will aide in the production and marketing of nutraceutical products based on *C. frondosa* viscera lipids.

## Figures and Tables

**Table 1:** Seasonal variation in total lipid and moisture content of viscera of *C. frondosa* (*n* = 3; mean ± (SD)). Total lipid and moisture are expressed relative to total mass. Fatty acid (FA) content is mass % of total FA in the lipid extract. Values in the same row with different letters are significantly different (*p* < 0.05).

Component	Winter		Spring		Summer	
Total lipid (wwb *)	7.36	(0.21) ^a^	4.93	(0.34) ^b^	5.30	(0.29) ^b^
Total lipid (dwb *)	28.87	(1.92) ^a^	20.81	(1.22) ^b^	23.83	(1.46) ^b^
Moisture content (%)	74.47	(1.09) ^b^	76.29	(0.08) ^a^	77.75	(0.37) ^a^
Total EPA (%)	24.74	(0.68) ^b^	25.38	(0.18) ^b^	28.23	(0.40) ^a^
Total PUFA (%)	27.31	(0.62) ^c^	28.03	(0.27) ^b^	29.72	(0.42) ^a^
12-MTA (%)	21.27	(0.77) ^a^	19.17	(0.93) ^b^	11.79	(0.78) ^c^
Total Branched FA (%)	31.13	(0.75) ^a^	27.84	(0.94) ^b^	18.21	(0.87) ^c^

* wwb = wet weight basis; dwb = dry weight basis.

**Table 2:** Proportions of FA (mass% total FA identified) in ether lipids isolated from *C. frondosa* viscera (*n* = 3; mean ± (SD)). Values with different letters (e.g., ^a–c^ for MAGE; ^e–g^ for DAGE) within a FA and lipid class are significantly different (ANOVA, Tukey’s test; *p* < 0.05).

	MAGE						DAGE					
	Winter		Spring		Summer		Winter		Spring		Summer	
4,8,12-Me-13:0	0.42	(0.00) ^b^	0.38	(0.02) ^c^	0.57	(0.05) ^a^	3.65	(0.05) ^e^	3.32	(0.19) ^f^	2.51	(0.34) ^g^
Me-14:0(a) *	0.10	(0.00) ^a^	0.09	(0.00) ^a^	0.11	(0.01) ^a^	0.85	(0.03) ^e^	0.84	(0.04) ^e^	0.80	(0.01) ^f^
Me-14:0(b) *	0.17	(0.03) ^b^	0.17	(0.02) ^b^	0.39	(0.04) ^a^	0.75	(0.01) ^e^	0.75	(0.02) ^e^	0.74	(0.04) ^e^
Me-14:0(c) *	0.08	(0.00) ^b^	0.06	(0.00) ^c^	0.11	(0.00) ^a^	0.67	(0.03) ^e^	0.57	(0.02) ^f^	0.45	(0.04) ^g^
12-MTA	4.81	(0.19) ^a^	4.95	(0.28) ^a^	5.79	(0.32) ^a^	42.21	(0.61) ^e^	41.09	(0.81) ^e^	37.12	(0.01) ^f^
8,12-Me-14:0	0.44	(0.03) ^a^	0.30	(0.03) ^b^	0.52	(0.02) ^a^	3.33	(0.45) ^e^	2.14	(0.29) ^f^	2.74	(0.14) ^g^
12-Me-15:0	2.09	(0.38) ^b^	2.31	(0.24) ^b^	3.98	(0.20) ^a^	7.06	(0.22) ^e^	6.84	(0.48) ^e^	5.19	(0.02) ^f^
8,12-Me-15:0	0.13	(0.04) ^ab^	0.11	(0.01) ^b^	0.19	(0.04) ^a^	0.48	(0.01) ^e^	0.44	(0.07) ^e^	0.31	(0.01) ^f^
ai-17:0	0.11	(0.01) ^ab^	0.09	(0.01) ^b^	0.13	(0.00) ^a^	0.56	(0.02) ^f^	0.57	(0.03) ^f^	0.77	(0.02) ^e^
14:0	0.16	(0.01) ^b^	0.17	(0.02) ^b^	0.56	(0.05) ^a^	0.53	(0.03) ^f^	0.61	(0.01) ^f^	1.40	(0.16) ^e^
15:0	0.03	(0.01) ^b^	0.13	(0.01) ^a^	0.00	(0.00) ^b^	0.23	(0.02) ^f^	1.37	(0.22) ^e^	0.00	(0.00) ^g^
16:0	0.76	(0.05) ^b^	0.75	(0.08) ^b^	3.15	(0.17) ^a^	0.68	(0.05) ^f^	0.72	(0.07) ^f^	1.99	(0.14) ^e^
18:0	0.99	(0.02) ^b^	0.92	(0.11) ^b^	2.43	(0.08) ^a^	0.50	(0.07) ^f^	0.49	(0.07) ^f^	1.14	(0.47) ^e^
20:0	0.03	(0.01) ^b^	0.05	(0.04) ^b^	0.19	(0.06) ^a^	0.04	(0.01) ^f^	0.08	(0.02) ^e^	0.03	(0.02) ^f^
22:0	0.09	(0.06) ^b^	0.14	(0.12) ^ab^	0.24	(0.00) ^a^	0.10	(0.04) ^f^	0.12	(0.02) ^ef^	0.16	(0.02) ^e^
16:1n-9	1.19	(0.45) ^ab^	1.39	(0.29) ^a^	0.90	(0.06) ^b^	0.15	(0.03) ^f^	0.17	(0.03) ^f^	0.27	(0.02) ^e^
16:1n-7	11.16	(0.32) ^b^	11.20	(0.32) ^b^	13.00	(0.47) ^a^	20.65	(0.51) ^e^	20.85	(0.30) ^e^	19.54	(0.82) ^f^
18:1n-9	0.48	(0.03) ^b^	0.44	(0.05) ^b^	1.02	(0.01) ^a^	0.86	(0.04) ^f^	0.96	(0.14) ^f^	2.16	(0.12) ^e^
18:1n-7	2.50	(0.14) ^b^	2.41	(0.29) ^b^	3.11	(0.08) ^a^	2.74	(0.06) ^e^	2.84	(0.17) ^e^	2.77	(0.11) ^e^
20:1n-11	0.21	(0.02) ^a^	0.22	(0.02) ^ab^	0.19	(0.01) ^b^	0.20	(0.01) ^g^	0.26	(0.03) ^f^	0.32	(0.01) ^e^
20:1n-9	0.44	(0.01) ^a^	0.43	(0.05) ^a^	0.53	(0.02) ^a^	0.29	(0.00) ^g^	0.40	(0.04) ^f^	0.53	(0.03) ^e^
20:1n-7	0.08	(0.01) ^b^	0.01	(0.01) ^c^	0.37	(0.01) ^a^	0.07	(0.01) ^f^	0.04	(0.00) ^g^	1.42	(0.51) ^e^
22:1n-9	0.14	(0.05) ^a^	0.12	(0.03) ^a^	0.19	(0.00) ^a^	0.09	(0.02) ^f^	0.14	(0.03) ^f^	0.26	(0.03) ^e^
22:1n-7	0.11	(0.04) ^a^	0.10	(0.02) ^a^	0.15	(0.00) ^a^	0.13	(0.02) ^f^	0.15	(0.02) ^ef^	0.19	(0.01) ^e^
24:1	1.29	(0.08) ^a^	1.32	(0.48) ^a^	1.06	(0.02) ^a^	0.62	(0.04) ^e^	0.65	(0.07) ^e^	0.62	(0.07) ^e^
EPA	61.79	(0.32) ^a^	62.26	(0.22) ^a^	53.17	(0.71) ^b^	5.27	(0.28) ^g^	6.29	(0.85) ^f^	9.10	(0.22) ^e^
DHA	1.06	(0.02) ^a^	1.12	(0.06) ^a^	1.08	(0.05) ^b^	0.30	(0.02) ^f^	0.39	(0.08) ^e^	0.43	(0.01) ^e^
Others	7.81	(0.43) ^a^	7.07	(0.44) ^a^	4.91	(0.69) ^b^	5.36	(0.15) ^e^	5.32	(0.22) ^e^	5.22	(0.37) ^e^
Sum	100		100		100		100		100		100	

* Me-14:0a, b and c are branched isomers; positions of the methyl branches were not determined. Note: 20:4n-6 was not reported because it could not be accurately quantified due to co-elution with an unidentified peak; total mass percent of the two co-eluting peaks was <2% in all lipid classes.

**Table 3:** Proportions of FA (mass% total FA identified) in PL and TAG isolated from *C. frondosa* viscera (*n* = 3; mean ± (SD)). Values with different letters (e.g., ^a–c^ for PL; ^e–g^ for TAG) within a FA and lipid class are significantly different (ANOVA, Tukey’s test; *p* < 0.05). The PL proportion included a small contribution from MAG.

	PL						TAG					
	Winter		Spring		Summer		Winter		Spring		Summer	
4,8,12-Me-13:0	0.08	(0.01) ^a^	0.06	(0.01) ^a^	0.10	(0.04) ^a^	3.98	(0.21) ^e^	4.11	(0.53) ^e^	2.87	(0.37) ^f^
Me-14:0(a) *	0.00	(0.00) ^b^	0.00	(0.00) ^b^	0.44	(0.03) ^a^	0.57	(0.04) ^e^	0.61	(0.03) ^e^	0.29	(0.03) ^f^
Me-14:0(b) *	0.14	(0.01) ^a^	0.14	(0.01) ^a^	0.06	(0.04) ^b^	0.52	(0.02) ^e^	0.55	(0.02) ^e^	0.42	(0.13) ^e^
Me-14:0(c)	0.02	(0.01) ^a^	0.00	(0.00) ^a^	0.03	(0.01) ^a^	0.30	(0.02) ^e^	0.28	(0.05) ^e^	0.12	(0.02) ^f^
12-MTA	0.46	(0.03) ^a^	0.32	(0.04) ^b^	0.45	(0.14) ^b^	19.15	(0.81) ^e^	19.87	(0.92) ^e^	9.99	(0.74) ^f^
8,12-Me-14:0	0.04	(0.02) ^b^	0.02	(0.01) ^b^	0.17	(0.06) ^a^	1.75	(0.25) ^e^	1.27	(0.12) ^f^	1.04	(0.08) ^g^
12-Me-15:0	0.21	(0.03) ^b^	2.39	(0.88) ^a^	3.58	(0.30) ^a^	2.56	(0.29) ^e^	2.51	(0.30) ^e^	1.33	(0.01) ^f^
8,12-Me-15:0	0.00	(0.00) ^b^	0.04	(0.01) ^a^	0.03	(0.00) ^ab^	0.18	(0.04) ^e^	0.17	(0.04) ^e^	0.06	(0.02) ^f^
ai-17:0	0.44	(0.09) ^a^	0.38	(0.00) ^a^	0.43	(0.21) ^a^	1.25	(0.07) ^g^	1.31	(0.01) ^e^	0.91	(0.02) ^f^
14:0	0.25	(0.11) ^a^	0.16	(0.02) ^b^	0.28	(0.11) _ab_	3.43	(0.07) ^g^	3.82	(0.10) ^f^	5.94	(0.25) ^e^
15:0	0.03	(0.02) ^a^	0.04	(0.01) ^a^	0.00	(0.00) ^b^	0.45	(0.24) ^f^	0.91	(0.28) ^e^	0.00	(0.00) ^g^
16:0	5.06	(0.24) ^b^	2.09	(0.31) ^c^	5.18	(1.06) ^a^	3.11	(0.07) ^g^	3.43	(0.13) ^f^	4.35	(0.32) ^e^
18:0	12.14	(0.65) ^a^	6.43	(0.13) ^b^	7.99	(1.11) ^b^	3.00	(0.06) ^f^	2.97	(0.10) ^f^	3.71	(0.44) ^e^
20:0	1.03	(0.09) ^a^	1.21	(0.02) ^a^	0.23	(0.07) ^b^	0.26	(0.02) ^f^	0.36	(0.01) ^e^	0.26	(0.02) ^f^
22:0	0.96	(0.05) ^b^	1.08	(0.04) ^b^	1.65	(0.24) ^a^	0.31	(0.01) ^ef^	0.35	(0.09) ^e^	0.25	(0.02) ^f^
16:1n-9	0.27	(0.08) ^c^	2.96	(0.85) ^a^	1.93	(0.26) ^b^	0.14	(0.01) ^f^	0.15	(0.01) ^ef^	0.17	(0.00) ^e^
16:1n-7	1.85	(0.24) ^b^	1.51	(0.06) ^c^	3.08	(0.07) ^a^	23.73	(0.41) ^f^	24.15	(0.31) ^f^	27.61	(0.62) ^e^
18:1n-9	1.17	(0.04) ^a^	1.31	(0.06) ^a^	1.77	(0.62) ^a^	1.99	(0.05) ^g^	2.18	(0.07) ^f^	2.75	(0.17) ^e^
18:1n-7	4.21	(0.42) ^a^	4.16	(0.25) ^a^	3.38	(0.24) ^b^	2.50	(0.04) ^ef^	2.79	(0.14) ^e^	2.34	(0.27) ^f^
20:1n-11	2.30	(0.08) ^a^	2.74	(0.06) ^a^	1.90	(0.47) ^b^	0.65	(0.05) ^f^	0.85	(0.04) ^e^	0.38	(0.04) ^g^
20:1n-9	1.03	(0.10) ^a^	1.02	(0.04) ^a^	1.77	(0.58) ^a^	0.60	(0.01) ^e^	0.70	(0.04) ^e^	0.63	(0.08) ^e^
20:1n-7	1.18	(0.18) ^a^	0.60	(0.13) ^b^	0.98	(0.33) ^b^	0.20	(0.08) ^f^	0.12	(0.01) ^f^	1.06	(0.23) ^e^
22:1n-9	1.20	(0.35) ^a^	1.20	(0.04) ^a^	1.16	(0.37) ^a^	0.47	(0.03) ^f^	0.57	(0.03) ^e^	0.48	(0.01) ^f^
22:1n-7	1.38	(0.17) ^b^	1.46	(0.04) ^b^	2.07	(0.06) ^a^	0.50	(0.03) ^f^	0.56	(0.03) ^e^	0.38	(0.02) ^g^
24:1	1.80	(0.15) ^a^	1.50	(0.12) ^c^	2.09	(0.04) ^b^	0.90	(0.07) ^e^	0.96	(0.05) ^e^	0.62	(0.19) ^f^
EPA	51.79	(2.82) ^a^	55.20	(0.72) ^a^	43.45	(0.43) ^b^	16.71	(0.20) ^f^	14.06	(0.82) ^g^	20.06	(0.41) ^e^
DHA	1.66	(0.24) ^a^	1.73	(0.11) ^a^	1.38	(0.33) ^b^	0.92	(0.07) ^e^	0.86	(0.03) ^e^	0.79	(0.02) ^f^
Others	8.04	(0.57) ^a^	9.01	(0.68) ^a^	11.57	(0.72) ^a^	5.76	(0.16) ^f^	5.73	(0.14) ^f^	6.22	(0.29) ^e^
Sum	100		100		100		100		100		100	

* Me-14:0a, b and c are branched isomers; positions of the methyl branches were not determined. Note: 20:4n-6 was not reported because it could not be accurately quantified due to co-elution with an unidentified peak; total mass percent of the two co-eluting peaks was <2% in all lipid classes.

## Data Availability

Full data is available from the authors on request.

## Supplementary Materials

The following supporting information can be downloaded at: https://www.mdpi.com/article/10.3390/md20070435/s1, Standards for HPLC and TLC; Table S1: Gradient elution system for lipid class profiling; Figure S1: Lipid class profiling of *C. frondosa* lipids by HPLC: (a) DAGE; (b) TAG; (c) FFA; (d) DAG; (e) MAG; and (f) PL. Figure S2: ^13^C-NMR (A) and ^1^H NMR (B) of recovered the MAGE band from *C. frondosa* viscera in CDCl_3_.
