# Supplementary material for: Unusual Ether Lipids and Branched Chain Fatty Acids in Sea Cucumber (Cucumaria frondosa) Viscera and Their Seasonal Variation
Source: Mar Drugs. 2022 Jun 29;20(7):435. doi: 10.3390/md20070435 (PMC9318488; doi:10.3390/md20070435)
Supplement: Supplementary file 1 [file marinedrugs-20-00435-s001.zip › marinedrugs-1775022-supplementary.pdf]

Supplementary Material

Standards for HPLC and TLC

The standards used for the HPLC analysis included free fatty acids (FFA; oleic acid, elaidic acid and 9-cis,12-cis-linoleic acid), 1-oleoyl-*rac*-glycerol (MAG), olive oil as a TAG standard, 1,2-dipalmitoyl-*sn*-glycerol (DAG), 1-*O*-hexadecyl-2,3-hexadecanoyl glycerol (diacylglyceryl ether; DAGE)), and a PL mixture containing L-α-lysophosphatidylcholine from *Glycine max* (soybean), L-α-phosphatidylcholine, L-α-phosphatidylethanolamine from *Glycine max* (soybean), and L-α-phosphatidylinositol sodium salt from *Glycine max* (soybean). The standards used in the TLC study were arachidic acid stearyl ester (wax ester; WE), 1-*O*-hexadecyl-2,3-hexadecanoyl glycerol (DAGE)), tristearin (TAG); 1,2-distearine (DAG), 1-monostearin (MAG), stearic acid (FFA), phosphatidyl choline dipalmitoyl (PC) and cholesterol (sterol).

Supplementary Table S1: Gradient elution system for lipid class profiling.

Time (min)	Solvent A (%)	Solvent B (%)	Solvent C (%)
0	100	0	0
12	99	1	0
16	98	2	0
25	94	6	0
40	92	8	0
44	75	25	0
48	20	80	0
52	0	100	0
62	0	50	50
68	0	15	85
70	0	0	100
72	0	0	100
74	0	100	0
77	90	10	0

Solvent A = 0.2 % v/v ethyl acetate in isooctane, Solvent B = 0.02 % v/v acetic acid in 2:1 v/v acetone: ethyl acetate and Solvent C = 0.1 % v/v acetic acid in 3:3:1 v/v/v isopropyl alcohol: methanol: water.

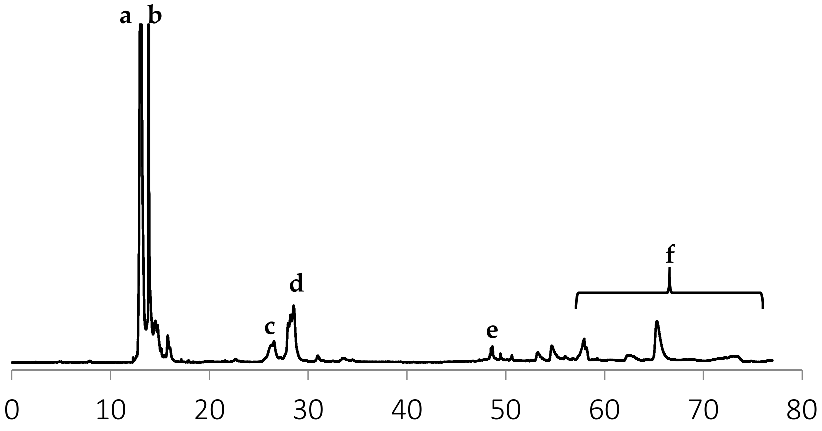

Supplementary Figure S1: Lipid class profiling of *C. frondosa* lipids by HPLC: a) DAGE; b) TAG; c) FFA; d) DAG; e) MAG; and f) PL.

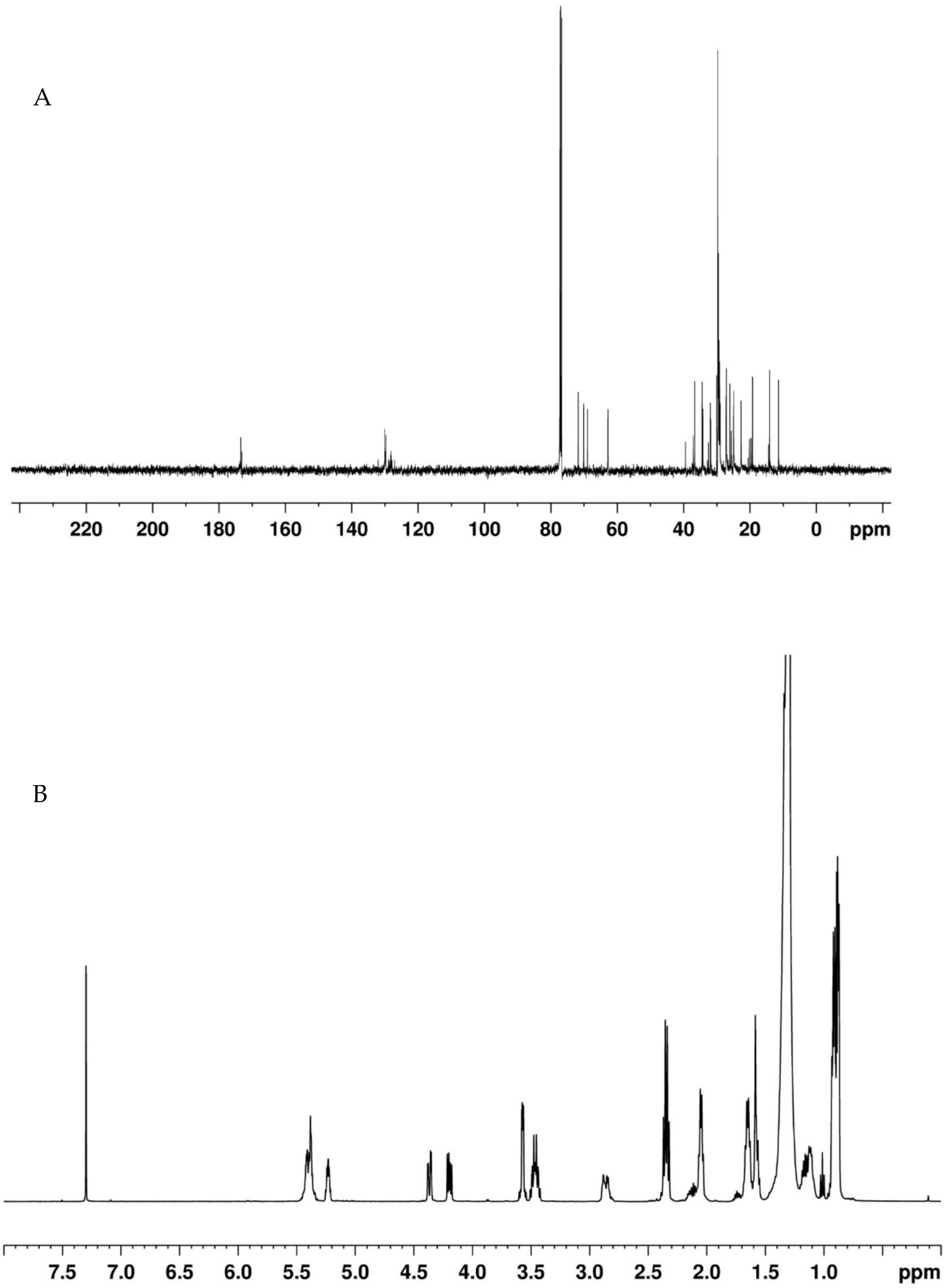

Supplementary Figure S2: ^{13}C-NMR (A) and ^{1}H NMR (B) of recovered the MAGE band from *C. frondosa* viscera in $CDCl_3$. Peaks at ~3.5 ppm in A, and 70-75 ppm in B support the presence of compounds containing ether bonds.
